# Cholesterol‐regulated cellular stiffness may enhance evasion of NK cell‐mediated cytotoxicity in gastric cancer stem cells

**DOI:** 10.1002/2211-5463.13793

**Published:** 2024-03-17

**Authors:** Lijuan Zhu, Hongjin Wang

**Affiliations:** ^1^ Department of Radiation Oncology (II) The First Affiliated Hospital of Jinzhou Medical University China; ^2^ Department of Obstetrics and Gynecology The First Affiliated Hospital of Jinzhou Medical University China

**Keywords:** cancer stem cells, cholesterol, gastric cancer, immunotherapy, NK cell, SREBP2

## Abstract

Gastric cancer has a high rate of recurrence, and as such, immunotherapy strategies are being investigated as a potential therapeutic strategy. Although the involvement of immune checkpoints in immunotherapy is well studied, biomechanical cues, such as target cell stiffness, have not yet been subject to the same level of investigation. Changes in the cholesterol content of the cell membrane directly influence tumor cell stiffness. Here, we investigated the effect of cholesterol on NK cell‐mediated killing of gastric cancer stem‐like cells. We report that surviving tumor cells with stem‐like properties elevated cholesterol metabolism to evade NK cell cytotoxicity. Inhibition of cholesterol metabolism enhances NK cell‐mediated killing of gastric cancer stem‐like cells, highlighting a potential avenue for improving immunotherapy efficacy. This study suggests a possible effect of cancer cell stiffness on immune evasion and offers insights into enhancing immunotherapeutic strategies against tumors.

## Graphical abstract

This research shows how gastric cancer stem‐like cells, through upregulation of SREBP2, elevate their cholesterol metabolism to evade natural Killer (NK) cytotoxicity. Targeting this metabolic pathway by inhibiting SREBP2‐related cholesterol synthesis markedly improves NK cell effectiveness against these cancer cells.

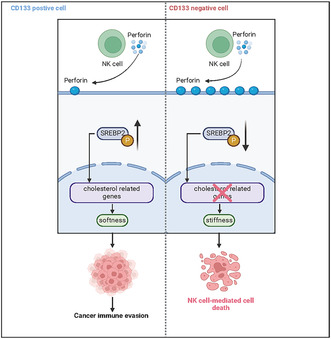

**Abbreviations**
5‐FU: 5‐fluorouracil (previously mentioned)
AFM: atomic force microscopy
CSC: cancer stem cell
DCs: dendritic cells
E : T ratio: effector‐to‐target ratio
F‐actin: filamentous actin
GC: gastric cancer
IL2, IL12, IL15, IL18: interleukins 2, 12, 15, and 18
MSCs: mesenchymal stromal cells
NK cell: natural killer cell
SREBP2: sterol regulatory element‐binding protein 2

Gastric cancer (GC) has the worst mortality risk among all malignancies [1, 2]. Despite advances in chemotherapy for GC, such as 5‐FU and platinum‐based regimens, chemotherapy effects remain unsatisfactory, with overall survival (OS) exceeding 2 years [3]. The high rate of recurrence is a result of accumulating resistance to conventional chemotherapy regimens and the absence of successful novel therapeutic approaches [4, 5, 6]. As a result, cancer immunotherapy strategies are being rigorously explored in clinical and preclinical studies [7, 8].

In the context of tumor immunotherapy, most studies have focused on biochemical signaling pathways as potential immune checkpoints, whereas biomechanical signals, such as target cell stiffness, have received less attention. Previous studies have demonstrated that the reduction in tumor cell stiffness is attributed to softening of the cell's cytoskeletal network and plasma membrane [9]. Cholesterol is an essential component of cell membranes that plays a wide range of physiological roles in the body [10]. Furthermore, changes in the cholesterol content of the cell membrane directly influence tumor cell stiffness [11]. The physical properties exhibited on the surface of target cells can potentially influence the dynamic interactions between tumor and T cells [12]. However, the role of cancer cell stiffness in the evasion of NK cell immune surveillance remains unclear. Understanding how target cell stiffness affects NK cell responses could provide valuable insights into tumor immune evasion and enhance the development of effective immunotherapy strategies.

In our study, we initially observed that NK cells were unable to eliminate all tumor cells. Furthermore, we found that the surviving cells exhibited cancer stem‐like properties and utilized upregulated cholesterol metabolism to evade NK cell‐mediated cytotoxicity. Moreover, inhibiting cholesterol metabolism effectively enhanced the NK cell‐mediated killing of gastric cancer stem‐like cells. Subsequently, we discovered that gastric cancer stem cells upregulate the transcription factor (sterol regulatory element‐binding protein 2) SREBP2. This upregulation promotes cholesterol metabolism, resulting in alterations in cell membranes, which makes them less susceptible to perforin released by NK cells. As a consequence, these tumor cells exhibit immune evasion, which further contributes to their enhanced survival and resistance against NK cell attacks.

## Materials and methods

### Ethics statement

The animal experiments performed in this study adhered to the rules set out by the Animal Ethics Committee and were authorized by the Institute Ethics Committee of Jinzhou Medical University (Approval No. 2022030211). All methods were performed in accordance with relevant guidelines and regulations. The study was conducted in accordance with the ARRIVE guidelines (https://arriveguidelines.org).

### Cell lines

MGC803 and AGS gastric tumor cell lines were acquired from the China Center for Type Culture Collection (Wuhan, China). These cell lines were cultured in RPMI 1640 medium (Thermo Fisher Scientific, Waltham, MA, USA, 11875093) supplemented with 10% fetal bovine serum (Thermo Fisher Scientific, 10270106) and 100 μg·mL^−1^ penicillin/streptomycin (Thermo Fisher Scientific, 10378016). The cell lines were cultured at a temperature of 37 °C in an environment containing 5% carbon dioxide. Human NK‐92 cells were acquired from the China Center for Type Culture Collection. The cells were cultured in alpha minimum essential medium (Thermo Fisher Scientific, 12571063) supplemented with specific concentrations of inositol (Selleck, Houston, TX, USA, S4530), 2‐mercaptoethanol (Sigma‐Aldrich, St. Louis, MO, USA, 60‐24‐2), folic acid (Sigma‐Aldrich, 59‐30‐3), and recombinant human IL‐2 (PeproTech, Rocky Hill, NJ, USA, 200‐02). The medium was further adjusted to a final concentration of 12.5% horse serum (Thermo Fisher Scientific, 26050088) and 12.5% fetal bovine serum (FBS) (Thermo Fisher Scientific).

### 3D gel culture

As previously mentioned, MCF‐7 and 4T1 cells were seeded in 3D Matrigel (Signal Transduct Target Ther. 2023; 8: 247.) In this study, 96‐well plates containing 50 μL of culture media and 50 μL of 3D gels were seeded with tumor cells (1 × 10^3^ cells per well). Three days were spent cultivating cells in the 3D gels. A Leica DM750 Microscope (Leica, Wetzlar, Germany) was used to examine the morphology of three‐dimensionally cultivated cells.

### *In vitro* cytotoxicity assays

Cytotoxicity was analyzed using the eBioscience™ Annexin V Apoptosis Detection Kit APC (Thermo Fisher Scientific, 88‐8007‐72), following the guidelines supplied by the manufacturer's instructions. In this study, target cells (consisting of 10 000 target cells) were subjected to co‐culture with NK‐92 cells at an effector‐to‐target (E : T) ratio of 2 : 1. This co‐culture was performed in 96‐well V‐bottom plates, with each well containing a total volume of 100 μL. The co‐culture process was conducted at a temperature of 37 °C for a duration of 6 h. Subsequently, the cytotoxicity of the co‐cultured cells was assessed.

### Chemicals

The antibodies and chemicals used during the current study were as follows: Anti‐SREBP2 antibody (Abcam, Cambridge, UK, ab30682), Perforin (Cell Signaling Technology, Danvers, MA, USA, #3693), GAPDH (Cell Signaling Technology, #5174), Anti‐mouse IgG, HRP‐linked Antibody (Cell Signaling Technology, #7076), Anti‐rabbit IgG, HRP‐linked Antibody (Cell Signaling Technology, #7074), and simvastatin obtained from MCE (HY‐17502). Recombinant human perforin protein (ab114201) was obtained from Abcam.

### Mice and mouse tumor model

The NOD‐SCID‐Il2rg^−/−^(NSG) mice (stock number: 005557) (6–8 weeks) mice used in this study were obtained from Charles River Laboratories (Beijing, China) and housed in a controlled environment that was free of pathogens and were raised at 21–25 °C, 50–60% humidity, and a 12 h light/12 h dark cycle with free access to food and water. The mice were provided with sterile food and water. In the stomach tumor model, NSG mice were injected with 1 × 10^6^ NK cells. Subsequently, NSG mice were subcutaneously injected with phosphate‐buffered saline (PBS) as controls or 20 mg·kg^−1^ of siRNA (either unconjugated or lipid‐conjugated) dissolved in phosphate‐buffered saline (PBS) at a volume of 160 μL. Tumor development was measured at regular intervals of 3 days using a caliper. There were six mice in each group. The volume of the tumor was determined by applying the formula: volume = 1/2 × length × width^2^. Mice were checked daily for any signs of illness. Upon visible or palpable cancer development or upon moribund appearance, mice were euthanized with a combination of CO_2_ and cervical dislocation to guarantee the death of the animals. After that, the tissues were collected for further analysis.

### Measurement of cell stiffness by AFM

Atomic force microscopy cell stiffness was measured according to standard methodology [13, 14]. AFM force curves were captured with a customized Dimension Icon AFM (Bruker) placed atop an IX81 inverted optical microscope (Olympus) that had a heating stage for live‐cell imaging and a ×20 objective. Using an XY stage, the materials were moved until the desired cell, which could be observed under an optical microscope, was positioned beneath the AFM tip. Using a PNP‐TR‐B cantilever (Nanoworld), the force curves on the cell were collected at a rate of ~ 5 μm·s^−1^ in the relative trigger mode (15 nm trigger threshold). By utilizing a thermal tuning and the deflection sensitivity of 170 nm·V^−1^, the cantilever spring constant was determined to be 0.08 N·m^−1^. Single cells were measured both before and after treatment at 37 °C. The force curves were processed using the bruker nanoscope analysis software (Billerica, MA, USA), which also computed the Young's modulus of the sample. This was accomplished by fitting the approach curve to an indentation of less than 500 nm (to account for stiffness) and assuming a cortical Poisson's ratio of 0.3.

### Preparation of siRNAs and transfection

The Human Sterol Regulatory Element‐Binding Protein 2 (SREBP2) small interfering RNA (siRNA) was acquired from Genechem, a company based in Shanghai, China. The Silencer™ siRNA Transfection II Kit (Thermo, AM1631) was used to transfect MGC803 or AGS cells to achieve knockdown of the target gene. Cells that underwent transfection were used for further tests after 48 h. In this study, we assessed the transfection efficiency of GC cells using quantitative polymerase chain reaction (qPCR).

### RT‐PCR

TRIzol (Invitrogen, Cat. 15596018, Carlsbad, CA, USA) was used to extract total RNA from cells which was then transcribed to cDNA using a high‐capacity cDNA reverse transcription kit (Applied Biosystems, Wilmington, DE, USA, Cat. 4368813). The nucleotide sequences of the synthetic oligonucleotides are shown in Table 1.

**Table 1:** Primer sequences for qRT‐PCR.

Gene	Sequence
h‐hmgcr‐f	ATAATCCTGGGGAAAATGCC
h‐hmgcr‐r	TCTTCTTGGTGCAAGCTCCT
h‐ldlr‐f	TCTTTACGTGTTCCAAGGGG
h‐ldlr‐r	TGCAGTTTCCATCAGAGCAC
INSIG1‐f	CCTGGCATCATCGCCTGTT
INSIG1‐r	AGAGTGACATTCCTCTGGATCTG
h‐hmgscr‐f	CTTCAGGTTCTGCTGCTGTG
h‐hmgcr‐r	CAGAAGAACTTACGCTCGGC
hNANOG‐f	TCCCGAGAAAAGATTAGTCAGCA
hNANOG‐r	AGTGGGGCACCTGTTTAACTT
hNESTIN‐f	CTGCTACCCTTGAGACACCTG
hNESTIN‐r	GGGCTCTGATCTCTGCATCTAC
hSOX2‐f	GCCGAGTGGAAACTTTTGTCG
hSOX2‐r	GGCAGCGTGTACTTATCCTTCT

### CD133 ^+^ tumor cells sorting

Tumor cells were collected by centrifugation at 800 revolutions per minute (rpm) for 5 min. Following PBS washing, the suspended cells were stained with PE‐conjugated anti‐CD133 antibody (BioLegend, San Diego, CA, USA, 372803) at room temperature for a duration of 15 min. Following the washing process, the cells were handled by filtration using a 40 μm cell strainer to create a single‐cell suspension. This suspension was then used for analysis or sorting using a BD FACSARIA III instrument manufactured by BD Bioscience (San Jose, CA, USA).

### Western blot

For western blot analysis, the extracts were lysed using RIPA buffer (Thermo Fisher Scientific), which contained phenylmethylsulfonylfluoride, sodium vanadate, and a protease inhibitor cocktail (Roche, Basel, Switzerland). Protein quantification was performed using a Bicinchoninic Acid Assay (Proteintech, Wuhan, China, PK10026). The proteins were denatured in RIPA buffer (Proteintech, PR20035) and subsequently loaded onto an SDS/PAGE gel (Proteintech, PR20004).

### Immunofluorescence assay

MGC803 or AGS cells were cultured on glass coverslips placed in 6‐well plates. Following various treatments, the cells were subjected to fixation using a 4% paraformaldehyde solution (Beyotime, Shanghai, China, P0099) for a duration of 60 min. Subsequently, the cells were treated with a 0.5% Triton X‐100 Solution (Beyotime, P0096) in PBS (Beyotime, ST447) for a period of 5 min. The cells were washed thrice with phosphate‐buffered saline (PBS) from Beyotime (ST447). Subsequently, the sections were blocked in QuickBlock buffer for 60 min at room temperature. Following this, The cells were incubated with an anti‐SREBP2 antibody (Abcam, ab30682) for a period of 20 h at a temperature of 4 °C. The cells were subjected to three washes in phosphate‐buffered saline (PBS) (Beyotime, ST447). Images were acquired using a fluorescence microscope (Leica Microsystems).

### Quantification of plasma membrane cholesterol levels

After fixing the gastric cancer cells with glutaraldehyde (0.1 weight percent in PBS), total cholesterol was extracted using methanol/chloroform (1 : 2, v/v), and the cells were sonicated. The Amplex Red cholesterol test kit was used to measure cholesterol levels after the organic solvent was removed under vacuum, following the previously mentioned procedure.

### Statistical analysis

Statistical analysis was conducted using spss (version 23.0; Armonk, NY, USA) and graphpad prism 9 (GraphPad, La Jolla, CA, USA). The predictive significance of SREBP2 expression in gastric cancer was assessed using the Kaplan–Meier plotter database (https://rookieutopia.com/). Data are reported as mean values accompanied by their corresponding standard deviations (SD). Student's *t*‐test and one‐way analysis of variance (more). Statistical significance was set at *P* < 0.05. All experiments were performed at least thrice.

## Result

### Gastric cancer stem cells evade NK cell‐mediated attack

**Fig. 1:**
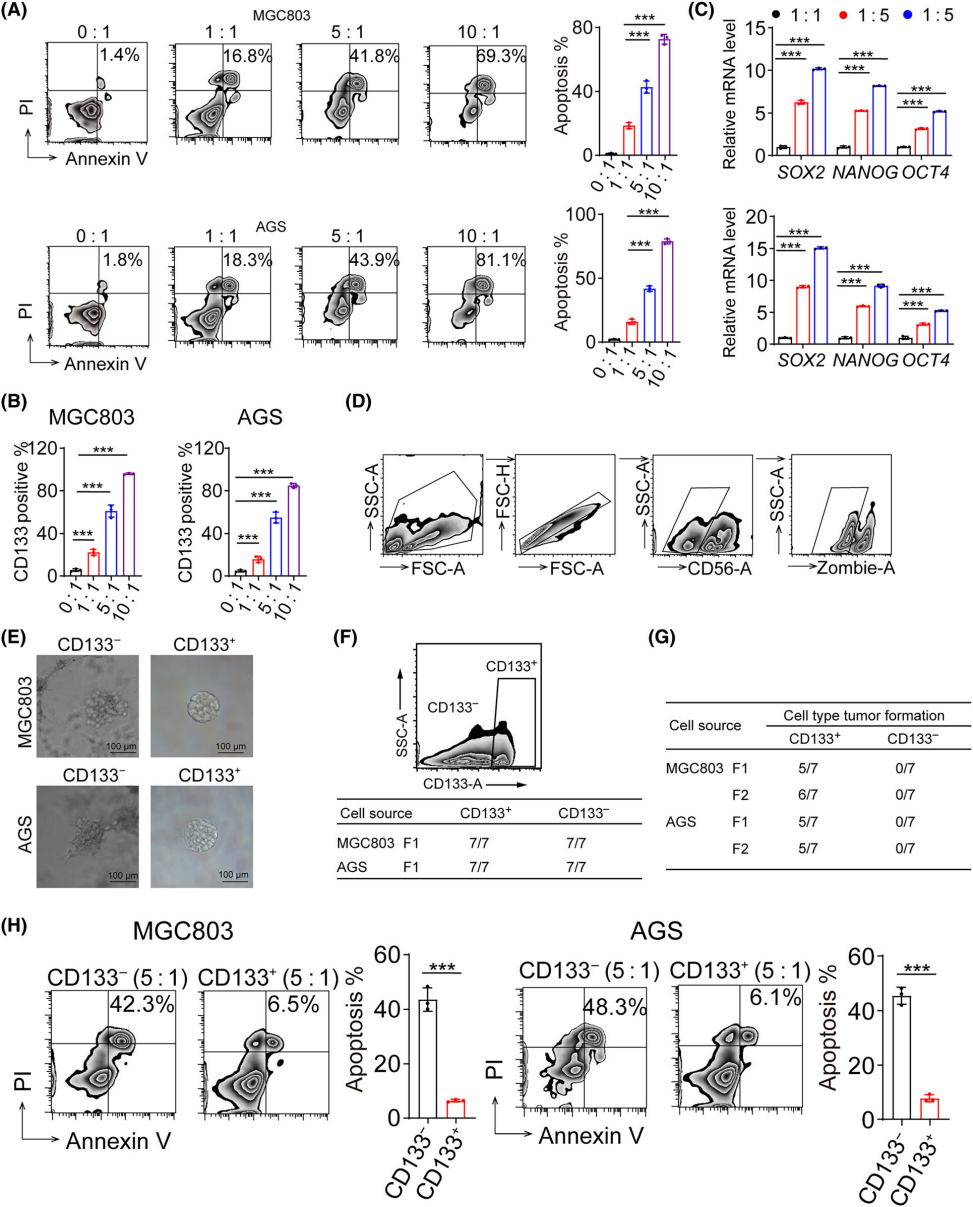
Gastric cancer stem cells evade NK cell‐mediated attack. (A) MGC803 or AGS cells were co‐incubated with NK‐92 at different ratios for 6 h. Cell apoptosis was determined using flow cytometry. (B) CD133 levels in MGC803 and AGS cells co‐incubated with NK‐92 cells at different E : T ratios were detected by flow cytometry. (C) NANOG, OCT4, and SOX2 levels in MGC803 and AGS cells co‐incubated with NK‐92 at different E : T ratios were detected using qPCR. (A) Surviving cancer cells from the NK tumor co‐culture system was determined by flow cytometry. (D) Surviving cancer cells from the NK tumor co‐culture system were determined by flow cytometry. (E) MGC803 (left) and AGS (right) cells were cultured in a flask system or 3D gel Matrigel for 3 days. *In vitro* colony formation assays, Bar 100 μm. (F) Flow cytometric analyzed expression of CD133 in tumors formed by CD133^+^ cells. (G) F1, and F2 generation cells were inoculated subcutaneously into NSG mice at a density of 5 × 10^4^ cells. We then analyzed tumor formation. (H) CD133^+^, CD133^−^ MGC803, or AGS cells were co‐incubated with NK‐92 for 6 h. Cell apoptosis was determined using flow cytometry. (A–D) *n* = 3 independent experiments. Two‐tailed Student's *t*‐test (H) or one‐way analysis of variance (ANOVA) followed by Bonferroni's test (A–D). The data are presented as the mean ± SD, *** represents *P* < 0.001.

The MGC803 and AGS gastric cancer cell lines were used to assess the cytotoxic effects of NK‐92 against gastric cancer. The cytotoxicity assay was performed using co‐cultures of effector (NK cells) and target (tumor cell) cells at various ratios. Despite the high efficacy of this treatment, some tumor cells died (Fig. 1A). Intriguingly, following NK cell killing, the CD133 level of surviving tumor cells was dramatically increased from 20% to 80%, accompanied by upregulation of SOX2, OCT4, and NANOG stem cell marker expression (Fig. 1B–D). To validate the resistance of cancer stem cells to NK cell attack, we performed cell sorting using a flow cytometer to separate CD133^+^ and CD133^−^ cell populations. According to previous studies [14], the 3D gel cell culture tumor sphere formation assay is a method used to assess the stemness of tumor cells. We found that CD133^+^ cells formed distinct clone spheres in the 3D gel, whereas CD133^−^ cells could not form noticeable tumor spheres (Fig. 1E). Moreover, we isolated CD133^+^ and CD133^−^ MGC803 and AGS gastric cancer cells (referred to as F0 cells) and inoculated them subcutaneously into NSG mice at a density of 5 × 10^4^ cells. We observed that approximately 5 out of 7 mice in the CD133^+^ group developed tumors, whereas none of the mice in the CD133^−^ group developed tumors. Flow cytometry analysis also revealed that the tumors derived from CD133^+^ F0 cells contained not only CD133^+^ tumor cells but also a significant proportion that had differentiated into CD133^−^ tumor cells (Fig. 1F). Furthermore, we isolated CD133^+/−^ tumor cells from F0 tumor tissues as F1 cells. Subsequently, we inoculated F1 CD133^+/−^ cells into new NSG mice and observed the same results as those of the F0 generation (Fig. 1G). This series of experiments indicated that the isolated CD133^+^ cells were cancer stem cells. Interestingly, CD133^+^ cells, which represent the cancer stem cell fraction, showed a greater ability to evade NK cell‐mediated cytotoxicity than CD133^−^ cells (Fig. 1H), which represent a non‐stem cell population, suggesting that a subpopulation of tumor stem cells evades NK cell killing.

### Cholesterol accumulation suppresses NK cell attack on gastric cancer stem cells

**Fig. 2:**
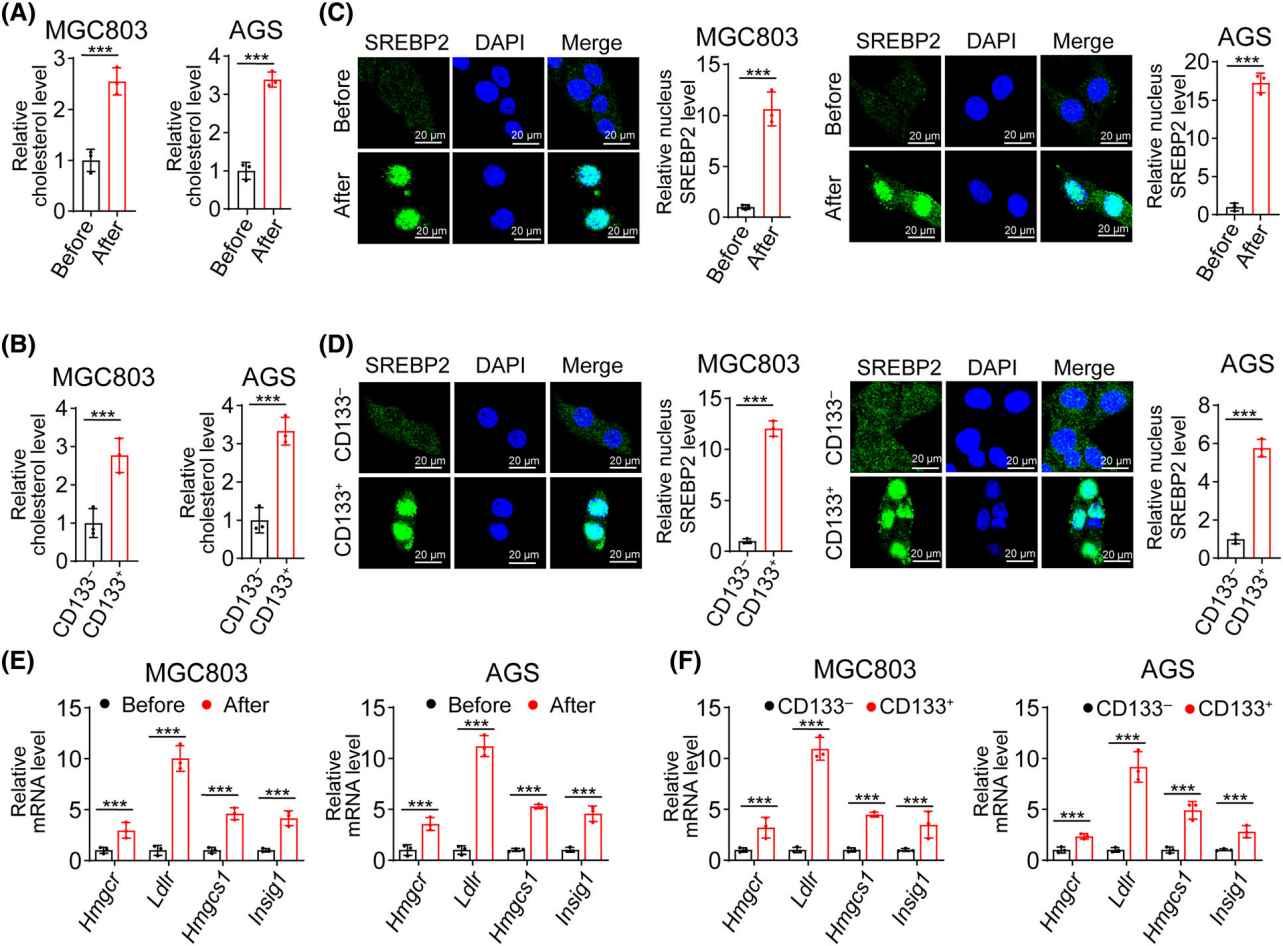
Cells resistant to NK cell‐mediated killing exhibit cholesterol accumulation. (A) Relative membrane cholesterol levels of MGC803 and AGS cells before (*n* = 3) or after (*n* = 3) co‐incubation were determined using a cholesterol assay kit. (B) CD133^high^ or CD133^−^ tumor cells were sorted by flow cytometry. The relative membrane cholesterol levels were also measured. (C) SREBP2 expression in MGC803 and AGS cells before or after co‐incubation was determined by immunofluorescence, Bar 20 μm. (D) CD133^high^ or CD133^−^ tumor cells were sorted by flow cytometry. SREBP2 levels were also measured, Bar 20 μm. (E) Gene expression in the cholesterol metabolism of MGC803 or AGS cells before or after co‐incubation was determined by qPCR. (F) CD133^high^ or CD133^−^ tumor cells were sorted by flow cytometry. Gene expression was detected during cholesterol metabolism. Two‐tailed Student's *t*‐test (A–D) or one‐way analysis of variance (ANOVA) followed by Bonferroni's test (E, F). The data are presented as the mean ± SD, *** represents *P* < 0.001.

**Fig. 3:**
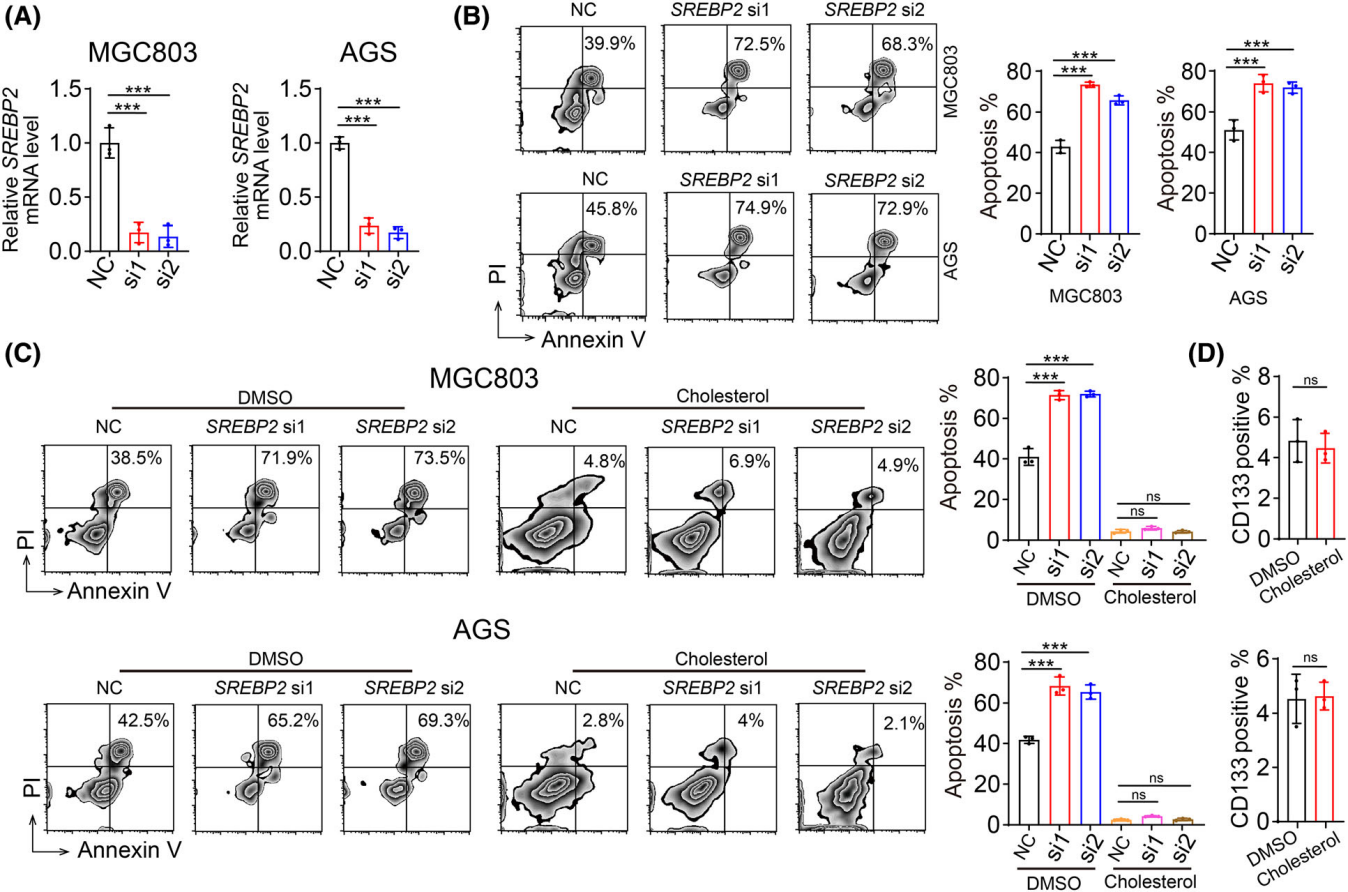
Cholesterol accumulation suppresses NK cell attack on gastric cancer stem cells. (A) Relative SREBP2 expression in MGC803 (left) and AGS (right) cells transfected with siNC and siSREBP2 was determined by qPCR. (B) NK92 cells were inoculated with siNC or si*SREBP2*‐MGC803/AGS cells, and an apoptosis assay was performed. (C) NK92 cells were inoculated with siNC or si *SREBP2*‐MGC803 or AGS cells, followed by cholesterol treatment (10 μm), and apoptosis assays were performed. (D) CD133 levels in MGC803 and AGS cells treated with cholesterol (10 μm) were detected by flow cytometry. (A–D) *n* = 3 independent experiments. Two‐tailed Student's *t*‐test (D) or one‐way analysis of variance (ANOVA) followed by Bonferroni's test (A–C). The data are presented as the mean ± SD. n.s no significant difference, *** represents *P* < 0.001, ns represents no significant difference.

Interestingly, we observed that the surviving cells, or CD133^+^ tumor cells (often considered as cancer stem cells), had higher cholesterol levels than cells prior to the cytotoxic event or bulk cells (Fig. 2A,B). SREBP2 is an enzyme involved in cholesterol synthesis [15, 16]. Remarkably, we also found elevated levels of SREBP2 in the surviving cells, or cancer stem cells, compared to cells before cytotoxicity or in bulk cells, and observed greater nuclear translocation of SREBP2 in the surviving cells, cancer stem cells compared to cells before cytotoxicity or CD133^−^ cells (Fig. 2C,D). Concurrently, we observed significant upregulation of enzymes related to cholesterol biosynthesis (Fig. 2E,F). Reducing cellular cholesterol levels by knocking out SREBP2 in gastric cancer stem cells significantly enhanced NK cell‐mediated cytotoxicity against these cells (Fig. 3A,B). Moreover, when we supplemented cholesterol after knocking out SREBP2 in gastric cancer stem cells, the cytotoxicity of NK cells against these cells was significantly reduced (Fig. 3C). Additionally, despite observing that CD133^high^ cells exhibited high expression of SREBP2, leading to an upregulation of cholesterol levels, supplementing additional cholesterol during the cell culture process did not increase the levels of CD133 (Fig. 3D). This indicated that cholesterol synthesis may be an intrinsic feature of cancer stem cells. In summary, these results suggest that gastric cancer stem cells may resist NK cell‐mediated cytotoxicity by activating SREBP2.

### Cholesterol decreases cancer cell rigidity and inhibits NK cell‐mediated cytotoxicity against gastric cancer

**Fig. 4:**
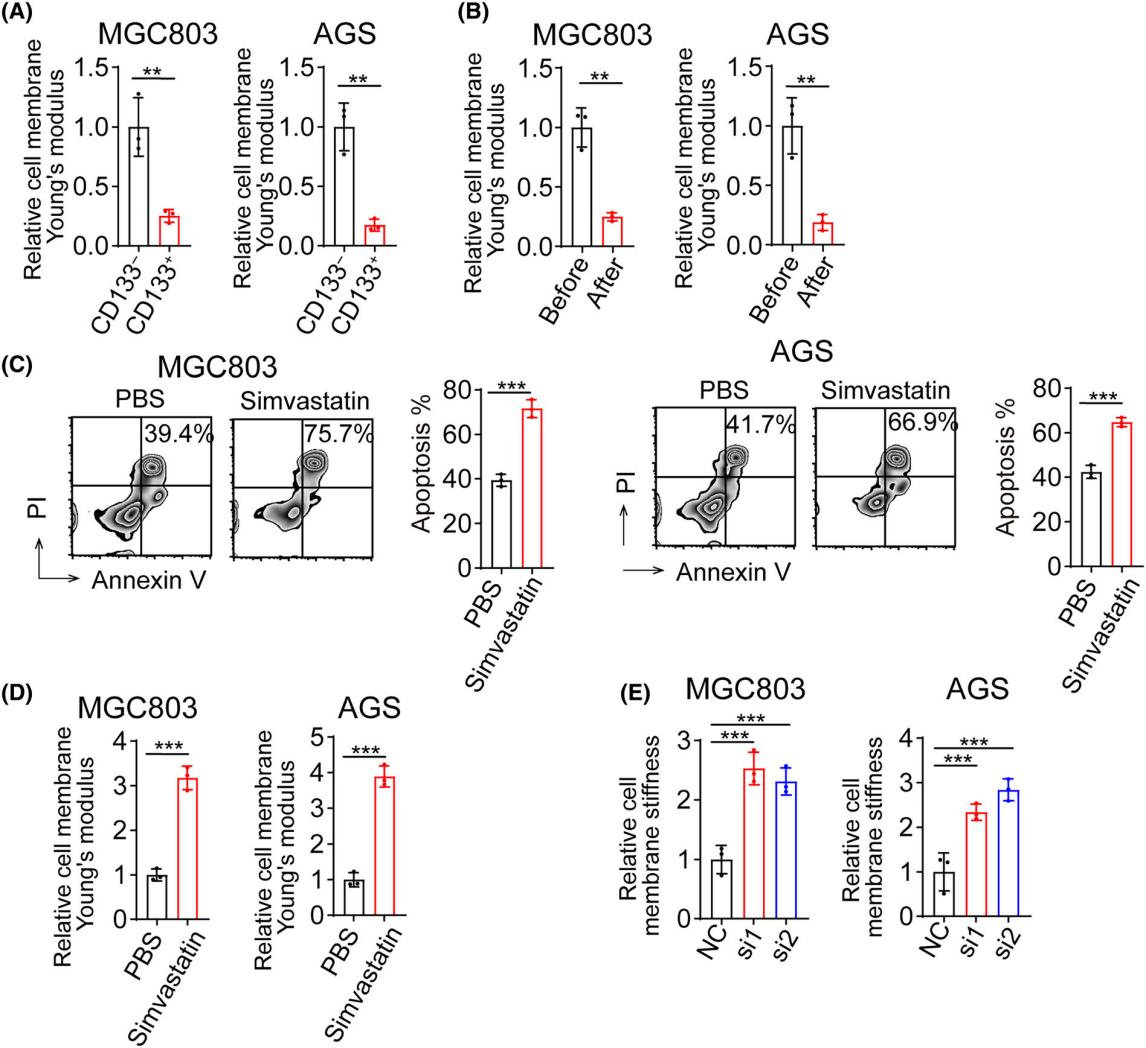
Cholesterol decreases cancer cell rigidity and inhibits NK cell‐mediated cytotoxicity against gastric cancer. (A) Relative cell membrane stiffness of CD133^high^ or CD133^−^ tumor cells sorted by flow cytometry. (B) Relative cell membrane stiffness of MGC803 and AGS cells before or after co‐incubation was determined by AFM. (C) Apoptosis levels of MGC803 (left) or AGS (right) cells treated with PBS, Simvastatin (10 μm), followed by co‐culture with NK92. (D) Relative cell membrane stiffness of MGC803 and AGS cells treated with PBS, Simvastatin (10 μm). (E) Relative cell membrane stiffness of MGC803 (left) or AGS (right) cells transfected with siNC and siSREBP2 determined by AFM. (A–E) *n* = 3 independent experiments. Two‐tailed Student's *t*‐test (A–D) or one‐way analysis of variance (ANOVA), followed by Bonferroni's test (E). The data are presented as the mean ± SD, ** represents *P* < 0.01, *** represents *P* < 0.001.

Previous studies have reported a strong correlation between cholesterol levels and cellular stiffness, and this factor may serve as a checkpoint for immune cell‐mediated cytotoxicity against tumor cells [17]. We isolated gastric cancer stem cells (CD133^+^) and non‐stem cells (CD133^−^) using flow cytometry and examined their cellular stiffness using atomic force microscopy. Interestingly, we found that cancer stem cells were significantly softer than non‐stem cells (Fig. 4A). Moreover, the cells that survived NK cell‐mediated cytotoxicity were noticeably softer than the cells before cytotoxicity (Fig. 4B). When we pretreated gastric cancer stem cells, which demonstrated resistance to NK cell‐mediated cytotoxicity, with simvastatin for 12 h before co‐culturing them with NK cells, we found that simvastatin treatment significantly sensitized these cells to NK cell‐mediated cytotoxicity compared to the PBS‐treated group, in line with significantly stiffened cells (Fig. 4C). Furthermore, we found that the knockdown of SREBP2 expression and simvastatin treatment in gastric cancer stem cells using siRNA significantly increased cellular stiffness (Fig. 4D,E).

### Cholesterol reduces the binding of perforin secreted by NK cells to target cells

**Fig. 5:**
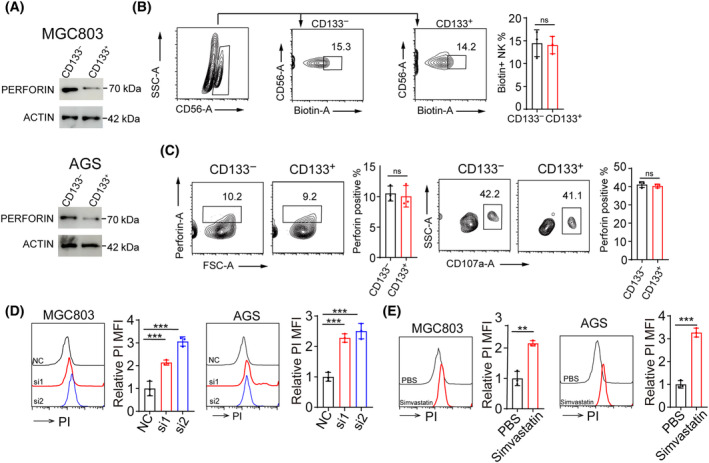
Cholesterol reduces the binding of perforin secreted by NK cells to target cells. (A) Relative Perforin levels of CD133^high^ or CD133^−^ tumor cells treated with PBS or recombinant human perforin protein. After washing with PBS three times, the tumor cells were extracted with membrane proteins, and perforin bound to the cell membrane was determined by western blotting. (B) Flow cytometric analysis of biotinylation in NK cells incubated with CD133^+^ or CD133^−^ cells. (C) Co‐culturing NK cells with CD133^+^ or CD133^−^ cells for 6 h. Perforin and CD107a of NK cells were analyzed by flow cytometry. (D) Relative PI levels of MGC803 (left) or AGS (right) cells transfected with siNC and siSREBP2, followed by treatment with PBS or recombinant human perforin protein were determined by flow cytometry. (E) Relative PI levels of MGC803 (left) or AGS (right) cells pretreated with PBS or Simvastatin, followed by treatment with PBS or recombinant human perforin protein were determined by flow cytometry. (B–E) *n* = 3 independent experiments. Two‐tailed Student's *t*‐test (B, C, E) or one‐way analysis of variance (ANOVA), followed by Bonferroni's test (D). The data are presented as the mean ± SD. n.s no significant difference, ** represents *P* < 0.01, *** represents *P* < 0.001, ns represents no significant difference.

**Fig. 6:**
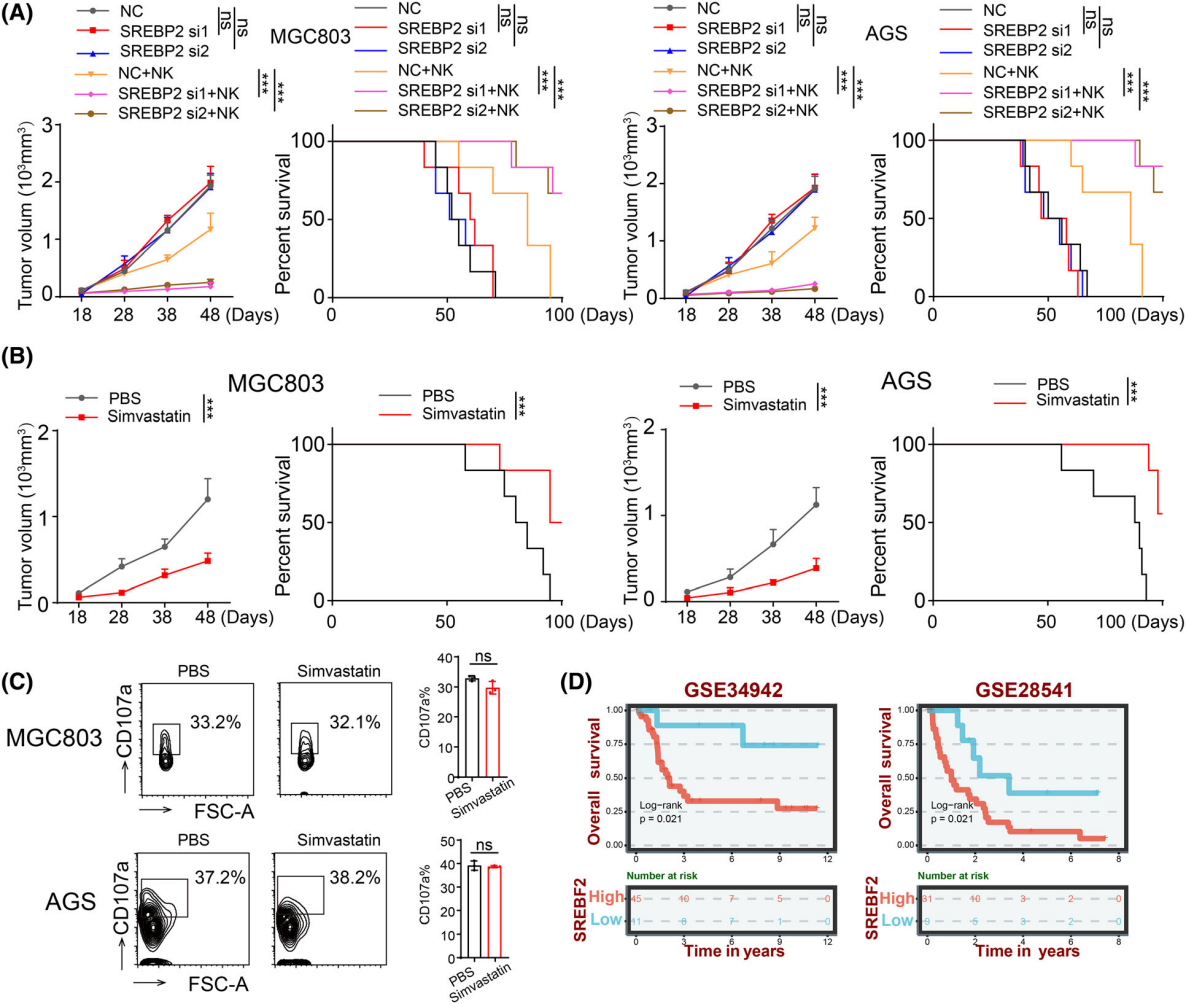
Cholesterol reduces NK cell‐mediated cytotoxicity *in vivo*. (A) NSG mice were inoculated with siNC‐ or *siSREBP2*‐MGC803 or AGS cells. Mice with 5 × 5 mm tumors were adoptively transferred with or without NK‐92 cells (1 × 10^6^ cells) once every 3 days for three times. Tumor growth and survival analyses were performed (*n* = 6). (B) NSG mice were inoculated with MGC803 or AGS cells. Mice with 5 × 5 mm tumor size were adoptively transferred with NK cells (1 × 10^6^ cells) once every 3 days three times, followed by treatment with PBS or Simvastatin once every 3 days. Tumor growth and survival analyses were performed (*n* = 6). (C) The experiment was the same as in (B), except that transferred NK cells were isolated from the tumors for flow cytometric analysis of the expression of CD107a. (D) Overall survival compared with SREBP2 levels in patients with gastric cancer. (C) or (F) *n* = 3 independent experiments. In (A), (B), *n* = 6 mice. Two‐tailed Student's *t*‐test (C) or one‐way analysis of variance (ANOVA), followed by Bonferroni's test (A, B) and log‐RANK (A, B, D). The data are presented as the mean ± SD. n.s no significant difference, *** represents *P* < 0.001, ns represents no significant difference.

We next sought to elucidate the molecular mechanisms by which cholesterol and cellular stiffness enable evasion of gastric cancer stem cells from NK cell‐mediated cytotoxicity. NK cells predominantly exert cytotoxicity against tumor cells through perforin and granzymes, with perforin forming pores on the target cell membrane to enable the ingress of granzymes, subsequently inducing cytotoxicity [18, 19]. Notably, we interestingly discovered that non‐stem tumor cells were more susceptible to perforin binding and pore formation than their cancer stem cell counterparts (Fig. 5A). After co‐culturing NK cells with CD133^+^ or CD133^−^ cells for 4 h, we employed FucoID technology to measure cell‐to‐cell interactions (*Cell*. 2020 Nov 12; 183(4): 1117–1133.e19). We also assessed the expression of CD107a and perforin in NK cells. We found no significant differences in the interactions between NK cells and CD133^+^ or CD133^−^ cells (Fig. 5B). Furthermore, we observed no differences in cytotoxic markers, including CD107a and perforin (Fig. 5C), suggesting that perforin‐mediated pore formation on the target cell membrane might be a key factor in the immune evasion exhibited by CD133^+^ cells. Nevertheless, when SREBP2 was knocked out or cells were treated with simvastatin, gastric cancer stem cells displayed an increased capability for pore formation (Fig. 5D,E). These results were validated *in vivo*. After SREBP2 knockout in tumor cells or simvastatin treatment, we observed that these interventions significantly promoted NK cell‐mediated cytotoxicity against gastric cancer *in vivo* and prolonged the survival of tumor‐bearing mice (Fig. 6A,B). Furthermore, simvastatin treatment did not affect the functionality of NK cells, implying its potential clinical value (Fig. 6C). Using a bioinformatics approach, we analyzed SREBP2 expression in patients with gastric cancer. The results showed that SREBP2 low expression was correlated with better survival in patients with gastric cancer. (Fig. 6D). These findings suggest that gastric cancer stem cells, by upregulating cholesterol biosynthesis, reduce their cellular stiffness, thereby preventing the perforin secreted by NK cells from forming pores in their cell membranes and consequently evading NK cell‐mediated immune attack.

## Discussion

NK cells have a high potential for anti‐tumor action [20]. NK cells kill tumor cells by releasing perforin/granzymes or triggering apoptotic pathways during antibody‐dependent or natural cytotoxicity [21]. NK cells maintain their anticancer activity and regulate other adaptive immune cell responses through the release of various cytokines [21]. NK cell function is regulated by a variety of cytokines, including IL2, IL12, IL15, and IL18, as well as by interactions with macrophages, dendritic cells (DCs), and mesenchymal stromal cells (MSCs) [22]. Thus, it is necessary to improve patient response to NK immunotherapy by discovering and targeting additional immunological checkpoints [20, 23, 24]. Here, we identified cellular stiffness as an immune checkpoint with a biomechanical element that cancer cells use to escape anti‐tumor immunity.

Several studies have explored the relationship between cholesterol metabolism and immune system. Previous studies have demonstrated how cholesterol metabolism affects the anti‐tumor activity of T cells [25], how cholesterol can act on immune cells to promote breast cancer metastasis and recurrence [25], and how elevated cholesterol levels induce endoplasmic reticulum stress, leading to increased XBP1 expression, which in turn promotes the expression of immune checkpoints and suppresses T cell function [26].

The interactions between tumors and immunity are complex and involve not only biochemical, but also significant biophysical signals. Our results suggest that SREBP2 KO CSC cells promote effective NK cell killing, and it is probable that cytoskeletal remodeling and membrane stiffness induced by cholesterol could contribute to this outcome. Stiffness is an intrinsic feature of cells and is mainly provided by F‐actin filaments and cholesterol [27]. Different cell types exhibit varying levels of stiffness, which correspond to the stiffness of the surrounding extracellular matrix and enable cells to recognize and respond efficiently to mechanical microenvironments [28]. Stiffness is a distinctive hallmark of gastric cancer that may provide insights into the fundamental biology of cancer, resulting in potential improvements in therapies and outcomes [29]. The mechanical aspect of cytotoxicity may have particular significance in anticancer immunity. Basu et al. observed that cytolytic secretory events predominantly occur within a domain that coincides with the region of highest force exertion [30]. Cancer stem cells from the primary tumor migrate away from this immunosuppressive environment during metastatic dissemination [31]. Notably, detached cancer cells are typically softer than their non‐transformed counterparts [32, 33, 34]. When they are not in the tumor microenvironment, this deformability may empower them to evade immune‐mediated attacks. Recently, it was demonstrated that the overexpression of myocardin‐related transcription factors improved cancer cell stiffness via filamentous actin rigidification and enhanced degranulation and cytokine production in cytotoxic CD8^+^ T lymphocytes [35]. Lei *et al*. suggested that different methods of stiffening cancer cells (membrane cholesterol depletion vs. intracellular cytoskeleton rigidification) may induce distinct T cell responses [9]. Nonetheless, since both the cell cortex and cytoskeleton contribute to cellular stiffness, modulating both components has the potential to overcome the mechanical immune checkpoint for enhanced cancer immunotherapy [27, 36].

In this study, we made a significant discovery regarding the evasion of NK cell immunotherapy by gastric cancer stem‐like cells. Through the activation of cholesterol metabolism mediated by SREBP2, these cells soften the cancer cells, thereby escaping NK cell attacks. To counteract this mechanism, we explored the control of cholesterol metabolism, such as by knocking out SREBP2 or promoting cell‐stiffening agents, in combination with NK immunotherapy. This approach resulted in a notable enhancement of NK cell cytotoxicity in gastric cancer cells. Consequently, in preclinical mouse tumor models, we observed tumor clearance and sustained responses, indicating promising therapeutic prospects. By targeting both metabolic and mechanical immune checkpoints in NK‐based immunotherapies, we envision the potential to benefit a broad range of patients.

## Conflict of interest

The authors declare no conflict of interest.

### Peer review

The peer review history for this article is available at https://www.webofscience.com/api/gateway/wos/peer‐review/10.1002/2211‐5463.13793.

## Author contributions

LZ and HW conceived and designed the study, and drafted the manuscript. LZ performed the experiments and conducted the data analysis.

## Data Availability

Data used to support the findings of this study are available from the corresponding author upon request.
